# Predicting postoperative pancreatic fistula in pancreatic head resections: which score fits all?

**DOI:** 10.1007/s00423-021-02290-x

**Published:** 2021-08-09

**Authors:** Mariam Adamu, Verena Plodeck, Claudia Adam, Anne Roehnert, Thilo Welsch, Juergen Weitz, Marius Distler

**Affiliations:** 1 Department of Visceral, Thoracic and Vascular Surgery, University Hospital Carl Gustav Carus, Technical University Dresden, Fetscherstraße 74, 01307 Dresden, Germany; 2 Department of Radiology, University Hospital Carl Gustav Carus, Technical University Dresden, Dresden, Germany

**Keywords:** Postoperative pancreatic fistula, Complications after pancreatic head resection, Risk score, External validation, Pancreatic surgery

## Abstract

**Purpose:**

Postoperative pancreatic fistula (POPF) is a major complication of pancreatic surgery and can be fatal. Better stratification of patients into risk groups may help to select those who might benefit from strategies to prevent complications. The aim of this study was to validate ten prognostic scores in patients who underwent pancreatic head surgery.

**Methods:**

A total of 364 patients were included in this study between September 2012 and August 2017. Ten risk scores were applied to this cohort. Univariate and multivariate analyses were performed considering all risk factors in the scores. Furthermore, the stratification of patients into risk categories was statistically tested.

**Results:**

Nine of the scores (Ansorge et al., Braga et al., Callery et al., Graham et al., Kantor et al., Mungroop et al., Roberts et al., Yamamoto et al. and Wellner et al.) showed strong prognostic stratification for developing POPF (*p* < 0.001). There was no significant prognostic value for the Fujiwara et al. risk score. Histology, pancreatic duct diameter, intraabdominal fat thickness in computed tomography findings, body mass index, and C-reactive protein were independent prognostic factors on multivariate analysis.

**Conclusion:**

Most risk scores tend to stratify patients correctly according to risk for POPF. Nevertheless, except for the fistula risk score (Callery et al.) and its alternative version (Mungroop et al.), many of the published risk scores are obscure even for the dedicated pancreatic surgeon in terms of their clinical practicability. There is a need for future studies to provide strategies for preventing POPF and managing patients with high-risk stigmata.

## Introduction

Postoperative pancreatic fistula (POPF) [1] is a major complication of pancreatic surgery. The main cause of POPF is insufficiency of the pancreatic anastomosis [2]. Although pain and sepsis are the main morbidity elements of POPF, mortality can occur due to serious adverse events like postpancreatectomy hemorrhage (PPH) [3]. Life-threatening PPH from the gastroduodenal or splenic artery stump, in the case of pancreatic head or tail resection respectively, has been documented [4, 5]. Advances in chemotherapy in the last decade have led to an enormous increase in the rate of pancreatic resections, mainly due to an increase in the proportion of patients with borderline resectable pancreatic ductal adenocarcinoma (PDAC) [6, 7].

The incidence of POPF has been shown to reach up to more than 30% [4, 5, 8–10] in a number of studies, even in high-volume pancreas centers. However, the management of patients in these high-volume centers plays a key role in the associated reduced mortality. Pancreatic fistulas are classified as biochemical leak, and grades B and C POPF according to the International Study Group of Pancreatic Surgery [11]. Grades B and C are considered clinically relevant POPFs, in which a change of management or reoperation is necessary and multiorgan failure or even death can occur. Benign tumors of the pancreas and chronic uncomplicated pancreatitis have an excellent prognosis. Thus, it is unacceptable to have an increased risk of mortality associated with postoperative complications due to POPF. Significant efforts have to be made to adequately predict a patient’s risk for developing POPF and ultimately identify patients who would benefit from additional approaches and closer perioperative monitoring. To improve the prognostic power of individual clinicopathological variables, several prognostic scoring systems have been proposed which combine multiple factors. To date, however, there is very limited data on the generalizability of these scoring systems.

Selection and stratification of patients into groups according to POPF risk are important for the application of additional surgical and clinical strategies in patient management. The aim of the present study was to select, validate and perform a head-to-head comparison of ten different prognostic (POPF) scoring systems in an independent cohort of patients with pancreatic head resection and prove their clinical practicability.

## Materials and methods

### Study population and data collection

This cohort study was carried out at the Department of Visceral, Thoracic and Vascular Surgery, University Hospital Carl Gustav Carus, Technical University Dresden. Patients who underwent either laparoscopic or conventional pylorus-preserving pancreaticoduodenectomy (PPPD), duodenum-preserving pancreatic head resection (DPPHR), or the Whipple procedure for chronic pancreatitis or benign or malignant lesions from September 2012 to August 2017 were prospectively enrolled in the study and their data included in a database. Patients with recurrent pancreatic surgery were excluded from the study. Follow-up examinations limited to this study were carried out at regular intervals and ended at month 3 after surgery. There was no loss to follow-up at 90 days after surgery.

At baseline, all participants completed a standard comprehensive questionnaire providing information on sociodemographic characteristics, medical history, current health status, and lifestyle factors. Data on diagnostic workup and tumor markers in the case of malignancy were collected from the hospital’s electronic database, patient files, and external reports. Information on patient follow-up was collected from the hospital’s electronic database. A visit to the special pancreatic consultation hour in our outpatient clinic takes place 3 months post-surgery at the latest. Patient follow-up took place until 90 days after surgery. This study was approved by the ethics committee of the Technical University Dresden (BO-EK-62022020) and performed according to the Declaration of Helsinki.

### Evaluation of prognostic scoring systems

Ansorge et al. [12] based their study on a cohort of 110 prospectively observed patients. The two prognostic factors included were pancreatic consistency (PC) and pancreatic duct diameter (PDD). Patients were assigned points according to these prognostic factors and stratified into 3 groups (“no risk,” “one risk factor,” and “two risk factors”). Braga et al. [13] performed a study on 700 patients, developed a score based on 469 of the patients, and validated it on 231. Predictors included in the scoring system were PC, PDD, operative blood loss, and American Society of Anesthesiologists (ASA) score. Patients were assigned points based on these risk factors and stratified into four groups according to their points. Similarly, Callery et al. [14] created a score based on PC, PDD, histology, and blood loss. The score was derived from 233 patients and validated prospectively on 212 patients. Outcomes were evaluated across 4 risk groups (“negligible risk,” 0 points; “low risk,” 1–2 points; “intermediate risk,” 3–6 points; “high risk,” 7–10 points). In a study from Japan, Fujiwara et al. [15] used different cutoff levels of albumin and C-reactive protein (CRP) to create a postoperative inflammatory score (PIS) which stratified patients into 3 groups (“PIS 0,” “PIS 1,” and “PIS 2”). Graham et al. [16] used age, body mass index (BMI), amylase level in drain on postoperative day two (POD 2) and PDD to estimate the probability of developing POPF. Kantor et al. [17] selected significant variables from a univariate analysis and included them in a multivariate analysis to create a model based on gender, BMI, preoperative total bilirubin, PDD, and gland texture. A 10-point model was created based on these variables. This model stratified patients into 4 groups (“negligible risk,” “low risk,” “intermediate risk,” and “high risk”). Mungroop et al. [18] created a final model of three strong predictors of POPF (soft pancreatic texture, decreasing PDD, and increasing BMI) based on the full model by Callery et al. [14] to predict the probability of POPF. In total, 2850 patients from 21 institutions from 4 countries were used for designing the model and its external validation. Roberts et al. [19] based their study on 325 patients. Using BMI and PDD, a risk score was derived from a model, which predicted the likelihood of developing POPF. In addition, a receiver operating characteristic (ROC) curve was produced and used to test the accuracy of the score. Wellner et al. [20] used preoperative variables, which included age, histology, history of smoking, weight loss, and pancreatitis, to stratify patients into “high-,” “medium-,” and “low-risk” groups. Yamamoto et al. [21] internally validated a score based on significant variables on univariate and multivariate analysis. The scoring system included main pancreatic duct (MPD), computed tomography findings on the relation of the portal vein (involved or away), gender, intraabdominal fat thickness [distance from the internal face of the rectus abdominis (linea alba) to the rear wall of the aorta at the level of the umbilicus], and histology of either pancreatic cancer or other diseases. Patients were assigned “0,” “1” or “2” points depending on the variables and the likelihood of developing POPF was estimated. An overview of the studies is presented in Table 1.

**Table. 1:** Overview of publications on the prognostic scores for postoperative pancreatic fistula

Author	Country	Published	Recruited	*N*	Stratification
Ansorge et al	Sweden	2012	2008–2010	110	no risk, one risk factor, two risk factors
Braga et al	Italy	2011	2002–2010	700^b^	0–3, 4–7, 8–11, 12–15
Callery et al	USA	2013	2002–2011	445^b^	negligible risk, low risk, intermediate risk, high risk
Fujiwara et al	Japan	2013	2001–2011	297^b^	PIS 0, PIS 1, PIS 2
Graham et al	USA	2013	2007–2012	146	probability estimate
Kantor et al	USA	2017	2011–2014	4827^b^	negligible risk, low risk, intermediate risk, high risk
Mungroop et al	Multiple^a^	2019	2007–2016	2850^b^	low risk, intermediate risk, high risk
Roberts et al	England	2014	2007–2012	325^b^	probability estimate
Wellner et al	Germany	2010	2006–2010	341^b^	low risk, medium risk, high risk
Yamamoto et al	Japan	2011	2004–2009	387^b^	0, 1, 2

^a^Netherlands, UK, Italy, USA

^b^Includes validation population

### Statistical analyses

A stepwise strategy was used to validate the selected risk scores. First, univariate analysis was performed including all variables in the selected scores. In order to reduce the standard error, Firth’s bias-reduced logistic regression was used for the univariate analysis. For the multivariate analysis, all significant univariate variables were considered and a stepwise backward selection procedure with penalized likelihood ratio test was used to select variables. The significance level for inclusion in the model was set at 0.05. The c-statistic was estimated in order to assess the predictive capacity of single models. The performance of the multivariate model was estimated by comparing the Akaike information criterion (AIC) of single factors included in the model and their combinations. In the descriptive analysis, continuous variables were expressed as median and interquartile range. Categorical variables were presented as number and percentage per group. The variables in the scores, on the other hand, were categorized as presented in the selected publications. For a better overview of the incidence of POPF, bar graphs were created where stratification into risk groups was available. Only patients with complete data on all variables were included in the respective statistical models. Statistical analyses were performed using the R 3.5.3 software [22].

## Results

### Patient characteristics

This cohort study included a total of 358 patients who underwent pancreatic head resection in the form of either the Whipple procedure, DPPHR, or PPPD (Table 2). The study comprised 145 (41%) women and 213 (59%) men. The median age of all patients was 66 years (interquartile range 56–74 years). Ninety-six percent of all patients underwent conventional surgery, 2% had minimally invasive surgery, and in another 2% conversion from minimally invasive to open surgery was required. The majority of patients (*n* = 245, 68%) underwent PPPD. More than 70% of the surgeries performed were due to malignancy. Clinically relevant POPF was diagnosed in 104 (29%) patients.

**Table. 2:** Overview of study population

	*n*	nav	%
Gender			
Female	145	358	41
Male	213	358	59
Age (years)			
Median (IQR)	66^a^	56^b^	74^b^
Surgery mode			
Conventional	345	358	96
Laparoscopic	7	358	2
Converted	6	358	2
Surgery type			
PPPD	245	358	68
Whipple operation	62	358	17
DPPHR	51	358	14
Histology			
Benign	103	358	29
Malignant	255	358	71
POPF grade			
None	239	358	67
Biochemical leak	15	358	4
B	54	358	15
C	50	358	14

*DPPHR*, duodenum-preserving pancreatic head resection; *IQR*, interquartile range; *n*, number; *nav*, number available; *POPF*, postoperative pancreatic fistula; *PPPD*, pylorus-preserving pancreaticoduodenectomy

^a^Median

^b^IQR

### Validation of prognostic scoring systems

We used a two-stage approach for external validation of the proposed scoring systems by performing uni- and multivariate analyses of the single variables included in the selected scores, followed by application of the calculated scores to patients operated at our clinic. In the ten prognostic scoring systems evaluated, 17 different clinicopathological variables were analyzed. On univariate analysis, stratification was performed according to the cutoff levels of individual scores (Table 3). A negative strength of association was seen for history of weight loss (OR 0.51, CI 0.32–0.82), history of pancreatitis (OR 0.43, CI 0.24–0.73), low postoperative CRP (OR 0.50, CI 0.29–0.85), and increase in PDD (OR 0.58, CI 0.49–0.68). A very high positive strength of association with the risk of developing POPF was seen in patients with a soft pancreatic consistency (OR 13.65, CI 6.65–30.83). Patients with a PDD < 3 mm had very high odds (OR 12.89, CI 6.60–26.92) of developing POPF. A medium positive strength of association was seen in patients with a histology other than PDAC or pancreatitis (OR 5.75, CI 3.53–9.53), and an MPD index < 0.25 (OR 5.50, CI 3.31–9.37). Lower ORs for developing POPF were estimated for distance of tumor from portal vein (OR 3.64, CI 1.32–13.77), a BMI $\ge$ 25 (OR 3.06, CI 1.91–4.94), histology other than PDAC (OR 2.86, CI 1.75–4.78), intraabdominal fat thickness > 6.5 cm (OR 2.66, CI 1.33–5.87), and amylase in drain (OR 1.06, CI 1.04–1.09).

**Table. 3:** Univariate analysis including all variables as presented in the scores and their association with POPF

Scores	*n*	nav	%	OR	95% CI		*p*	*n* analyzed
Mungroop et al. (2019, ref. 18)								211
PDD (truncated at 5 mm)								
Median (IQR)	4^d^	2^e^	5^e^	0.46	0.37	0.56	< 0.001	
Gland texture								
Firm	113	228	50					
Soft	115	228	50	13.65	6.65	30.83	< 0.001	
BMI		364	100					
Median (IQR)	24.61^d^	22.13^e^	27.72^e^	1.16	1.10	1.23	< 0.001	
Kantor et al. (2017, ref. 17)								211
Gender								
Female	147	364	40					
Male	217	364	60	1.59	0.99	2.58	0.05	
BMI								
< 25	200	364	55					
≥ 25	164	364	45	3.06	1.91	4.94	< 0.001	
Bilirubin^a^								
≥ 2 mg/dl	85	364	23					
< 2 mg/dl	279	364	77	1.65	0.94	3.00	0.08	
PDD								
≥ 6 mm	105	343	31					
3– < 6 mm	133	343	39	3.88	1.87	8.81	< 0.001	
< 3 mm	105	343	31	11.50	5.54	26.21	< 0.001	
Gland consistency								
Firm/intermediate	113	228	50					
Soft	115	228	50	13.65	6.65	30.83	< 0.001	
Roberts et al. (2014, ref. 19)								337
BMI								
Median (IQR)	24.61^d^	22.13^e^	27.72^e^	1.16	1.10	1.23	< 0.001	
PDD (mm)								
median (IQR)	4^d^	2^e^	6^e^	0.58	0.49	0.68	< 0.001	
Ansorge et al. (2012, ref. 12)								211
Pancreatic consistency								
1/2	113	228	50					
3/4	115	228	50	13.65	6.65	30.83	< 0.001	
PDD (mm)								
> 4	151	343	44					
3–4	87	343	25	7.02	3.48	14.95	< 0.001	
< 3	102	343	30	12.89	6.60	26.92	< 0.001	
< 2	3	343	1	6.60	0.57	53.74	0.11	
Callery et al. (2013, ref. 14)								137
Gland texture								
Firm	113	228	50					
Soft	115	228	50	13.65	6.65	30.83	< 0.001	
Histology								
PDAC/pancreatitis	209	280	75					
Other^b^	71	280	25	5.01	2.70	9.38	< 0.001	
PDD (mm)								
≥ 5	151	343	44					
4	39	343	11	4.77	1.92	11.87	< 0.001	
3	48	343	14	9.34	4.24	21.50	< 0.001	
2	102	343	30	12.89	6.60	26.92	< 0.001	
≤ 1	3	343	1	6.60	0.57	53.74	0.11	
Blood loss (ml)								
≤ 400	97	311	31					
401–700	103	311	33	0.75	0.39	1.41	0.37	
701–1000	56	311	18	1.46	0.72	2.94	0.29	
> 1000	55	311	18	1.36	0.66	2.77	0.41	
Fujiwara et al. (2013, ref. 15)								310
Albumin^c^								
High	187	321	58					
Low	134	321	42	0.77	0.47	1.26	0.30	
CRP^c^								
High	76	350	22					
Low	274	350	78	0.50	0.29	0.85	0.01	
Graham et al. (2013, ref. 16)								231
BMI		364	100					
Median (IQR)	24.61^d^	22.13^e^	27.72^e^	1.16	1.10	1.23	< 0.001	
Age		364	100					
Median (IQR)	66^d^	56^e^	74^e^	1.01	0.99	1.03	0.23	
Amylase in drain (umol/s*l)		249	68					
Median (IQR)	1.31^d^	0.16^e^	5.86^e^	1.06	1.04	1.09	< 0.001	
PDD (mm)								
≥ 3	238	343	69					
< 3	105	343	31	4.77	2.89	7.93	< 0.001	
Braga et al. (2011, ref. 13)								181
PDD (mm)								
> 3	190	343	55					
≤ 3	153	343	45	7.24	4.29	12.59	< 0.001	
Blood loss (ml)								
< 700	181	311	58					
≥ 700	130	311	42	1.64	1.00	2.71	0.05	
ASA								
I	10	361	3					
II	156	361	43	0.37	0.10	1.44	0.14	
III	195	361	54	0.60	0.17	2.32	0.45	
Yamamoto et al. (2011, ref. 21)								326
MPD index								
≥ 0.25	187	343	55					
< 0.25	156	343	45	5.50	3.31	9.37	< 0.001	
Distance from portal vein								
Involved	30	349	9					
Away	319	349	91	3.64	1.32	13.77	0.01	
Histology								
Pancreatic cancer	156	362	43					
Other	206	362	57	2.86	1.75	4.78	< 0.001	
Intraabdominal fat thickness (mm)								
≤ 65	61	346	18					
> 65	285	346	82	2.66	1.33	5.87	< 0.001	
Gender								
Female	147	346	40					
Male	217	346	60	1.59	0.99	2.58	0.05	
Wellner et al. (2010, ref. 20)								342
≤ 66 years	189	364	52					
> 66 years	175	364	48	1.29	0.82	2.04	0.27	
Histology								
Carcinoma/pancreatitis	220	362	61					
Other	142	362	39	5.75	3.53	9.53	< 0.001	
History of smoking								
No	246	360	68					
Yes	114	360	32	0.61	0.36	1.01	0.06	
Weight loss								
No	153	355	43					
Yes	202	355	57	0.51	0.32	0.82	< 0.001	
History of acute pancreatitis								
No	254	363	70					
Yes	109	363	30	0.43	0.24	0.73	< 0.001	

*ASA*, American Society of Anesthesiologists; *BMI*, body mass index; *CI*, confidence interval; *CRP*, C-reactive protein; *IQR*, interquartile range; *MPD*, main pancreatic duct; *n*, number; *nav*, number available; *OR*, odds ratio; *p*, *p* value; *PDAC*, pancreatic ductal adenocarcinoma; *PDD*, pancreatic duct diameter; *POPF*, postoperative pancreatic fistula; *ref*., reference number

^a^Preoperative bilirubin

^b^Ampullary, duodenal, cystic, islet cell

^c^Postoperative

^d^Median

^e^IQR %, percentage

On multivariate analysis, histology other than PDAC or pancreatitis (OR 3.98, CI 2.17–7.44), intraabdominal fat thickness > 6.5 cm (OR 2.67, CI 1.01–7.13), PDD according to the Callery et al. stratification (OR reaching 11.30 for PDD > 1–2 mm), and higher BMI (OR 1.08, CI 1.01–1.16) were strongly associated with POPF. Low postoperative CRP (OR 1.06, CI 1.04–1.09) had a negative strength of association with incidence of POPF (Table 4).

**Table. 4:** Significant variables on multivariate analysis including 302 patients

Score	Variable	OR	95% CI		*p*
Wellner et al	Histology of other than PDAC or pancreatitis	3.98	2.17	7.44	< 0.001
Yamamoto et al	Intraabdominal thickness > 65 mm	2.67	1.09	7.13	0.03
Callery et al	PDD 4 mm	6.48	2.20	19.32	< 0.001
	PDD 3 mm	7.98	3.22	20.78	< 0.001
	PDD 2 mm	11.30	5.21	26.40	< 0.001
	PDD ≤ 1 mm	5.41	0.38	55.74	0.19
Fujiwara et al	CRP^a^	0.44	0.21	0.88	0.02
Graham et al	BMI	1.08	1.01	1.16	0.03

*BMI*, body mass index; *CI*, confidence interval; *CRP*, C-reactive protein; *OR*, odds ratio; *p*, *p* value; *PDAC*, pancreatic ductal adenocarcinoma; *PDD*, pancreatic duct diameter

^a^Postoperative low

A two-step approach was used to estimate the performance of the multivariate model. First, the AIC values of single factors in a score were estimated and compared to those of the respective scores. In a number of scores, the AIC values of single factors were lower than that of the respective score model, meaning that the scores were unnecessarily complex for predicting POPF (data not shown). Furthermore, the AIC values of all single factors of the multivariate model were estimated and compared to that of the multivariate model (Table 5). As illustrated, it can be concluded that in some scores, single factors might be sufficient to predict POPF, because they have a better model fit than the score itself. However, our multivariate model has the perfect fit for this cohort and therefore, the lowest AIC of 259 compared to all single variables.

**Table. 5:** Akaike information criterion of single factors and the multivariate model

Score	Variable	AIC
Wellner et al	Histology of other than PDAC or pancreatitis	329
Yamamoto et al	Intraabdominal thickness > 65 mm	369
Callery et al	PDD 4 mm	305
Fujiwara et al	CRP^a^	367
Graham et al	BMI	347
Multivariate model probability		259

*AIC*, Akaike information criterion; *BMI*, body mass index; *CRP*, C-reactive protein; *PDAC*, pancreatic ductal adenocarcinoma; *PDD*, pancreatic duct diameter

^a^Postoperative low

**Fig. 1:**
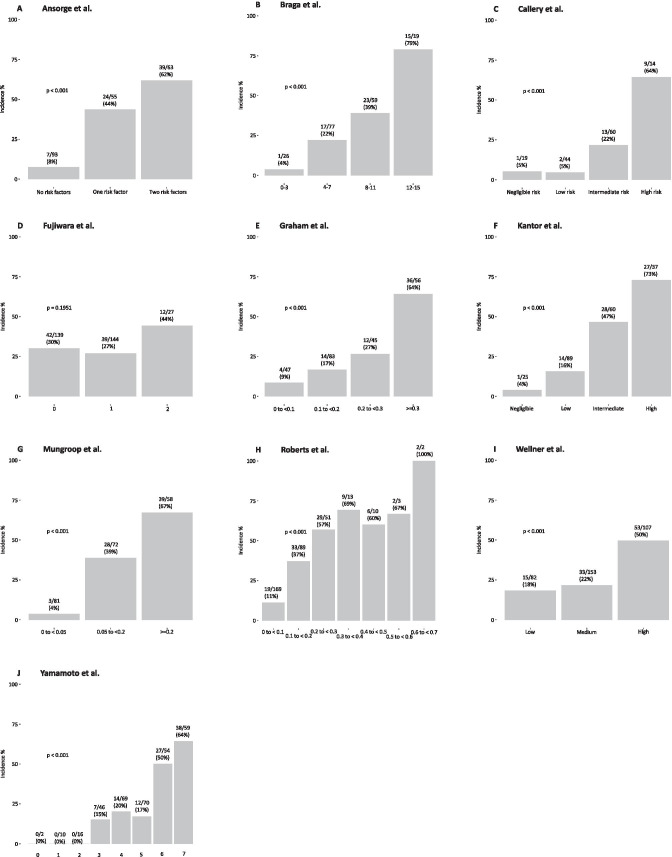
**A**—Bar graph demonstrating incidence of POPF according to Ansorge et al. risk stratification. POPF, postoperative pancreatic fistula. **B**—Bar graph demonstrating incidence of POPF according to Braga et al. risk stratification. POPF, postoperative pancreatic fistula. **C**—Bar graph demonstrating incidence of POPF according to Callery et al. risk stratification. POPF, postoperative pancreatic fistula. **D**—Bar graph demonstrating incidence of POPF according to Fujiwara et al. risk stratification. POPF, postoperative pancreatic fistula. **E**—Bar graph demonstrating incidence of POPF according to Graham et al. probability estimation. POPF, postoperative pancreatic fistula. **F**—Bar graph demonstrating incidence of POPF according to Kantor et al. risk stratification. POPF, postoperative pancreatic fistula. **G**—Bar graph demonstrating incidence of POPF according to Mungroop et al. probability estimation. POPF, postoperative pancreatic fistula. **H**—Bar graph demonstrating incidence of POPF according to Roberts et al. probability estimation. POPF, postoperative pancreatic fistula. **I**—Bar graph demonstrating incidence of POPF according to Wellner et al. risk stratification. POPF, postoperative pancreatic fistula. **J**—Bar graph demonstrating incidence of POPF according to Yamato et al. risk stratification. POPF, postoperative pancreatic fistula

**Fig. 2:**
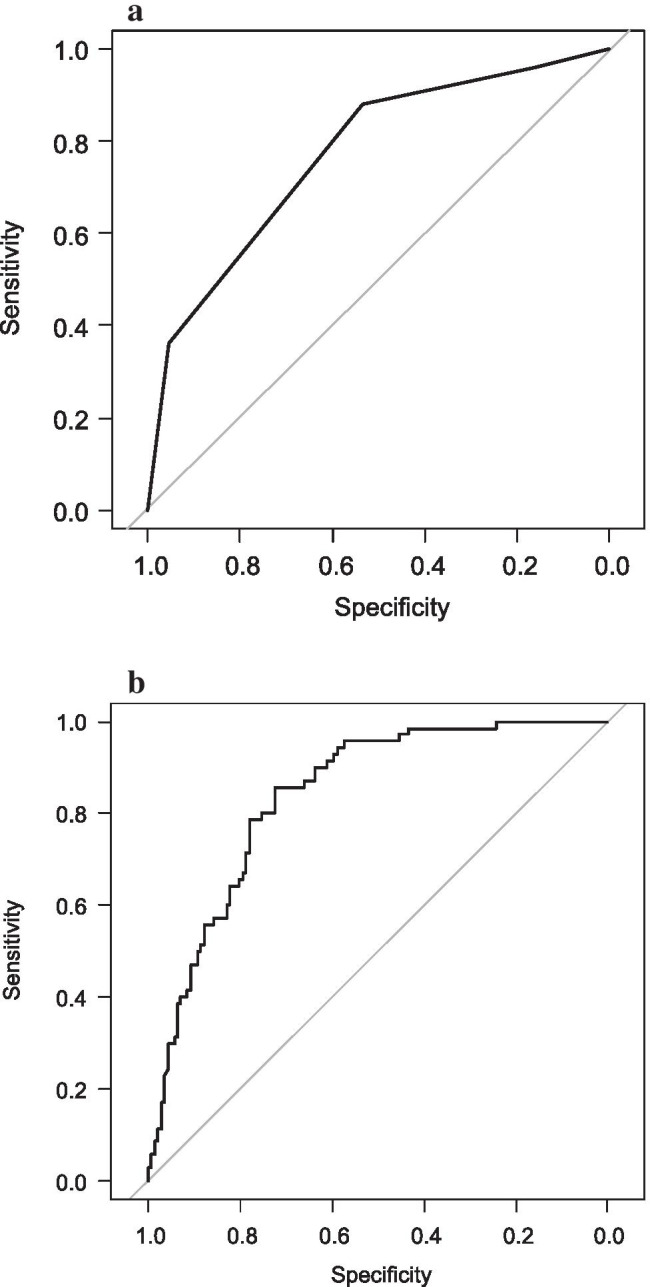
**A**—ROC curve for POPF, comparison to Callery et al. C-statistic (AUC) = 0.80, *p* = 0.00, (CI 0.68–0.91). **B**—ROC curve for POPF, comparison to Mungroop et al. C-statistic (AUC) = 0.84, *p* = 0.00, (CI 0.79–0.89). AUC, area under the curve; POPF, postoperative pancreatic fistula; ROC, receiver operating characteristic

Figure 1(A–J) shows an overview of the ten risk scores according to the different definitions. An adequate risk stratification was seen when the scores of Ansorge et al., Braga et al., Callery et al., Graham et al., Kantor et al., Mungroop et al., Roberts et al., Yamamoto et al., and Wellner et al. were applied to our cohort (*p* < 0.001). There was no significant prognostic value for the Fujiwara et al. risk score (*p* = 0.195). The predictive capacity of the Callery et al. and Mungroop et al. models for developing POPF was estimated as shown in Fig. 2(A–B). An overview of the model performance of each score is shown in Table 6.

**Table. 6:** Overview of the model performance of the selected scores

Score	AUC	CI	
Ansorge et al	0.79	0.73	0.85
Braga et al	0.74	0.67	0.81
Callery et al	0.80	0.68	0.91
Fujiwara et al	0.51	0.45	0.58
Graham et al	0.76	0.68	0.83
Kantor et al	0.82	0.76	0.88
Mungroop et al	0.84	0.79	0.89
Roberts et al	0.76	0.71	0.81
Wellner et al	0.68	0.62	0.75
Yamamoto et al	0.76	0.70	0.82

*AUC*, area under the curve; *CI*, confidence interval

## Discussion

To the authors’ knowledge, this is the first study to perform a head-to-head comparison and externally validate ten different published risk scores for POPF in an independent cohort of patients who underwent pancreatic head resection. On univariate analyses, several clinicopathological variables such as BMI, PDD, gland texture, histology, history of pancreatitis, MPD index, involvement of portal vein, intraabdominal thickness, and amylase in drain showed a strong association with POPF. A negative association with POPF was seen for history of pancreatitis, low postoperative CRP, and weight loss. No significant association with POPF could be found for multiple variables including gender, preoperative bilirubin, age at surgery, history of smoking, blood loss, albumin, and ASA classification. In line with these findings, application of the evaluated clinical risk scores to our patient population revealed a strong and clinically relevant stratification of patients’ risk for POPF by the scores of Ansorge et al., Braga et al., Callery et al., Graham et al., Kantor et al., Mungroop et al., Roberts et al., Yamamoto et al., and Wellner et al. There was no significant value for the Fujiwara et al. risk score.

As illustrated by most of the scores validated in this article, correct stratification of each patient according to risk factors is necessary in order to accurately predict the risk of POPF. Although the stratification of variables in the different scores differed, the tendency to predict risk of POPF was consistent across the different scores for the same variables stratified differently. This is also graphically depicted in the figures, which show an increase in incidence of POPF as the score value increases. While the majority of the variables included in the scores are easy to obtain, some can only be acquired intra- or postoperatively. It is not always the case that PDD, gland texture, and histology type are determined preoperatively. On the other hand, blood loss can only be determined during or after surgery. CT findings and biopsy (when indicated) can help determine some of the above-mentioned variables. Although CT findings are proximity measures, these measures are reproducible. The only subjective measure may be tumor infiltration of the portal vein. It is nevertheless challenging to stratify patients correctly preoperatively and discuss the risk of POPF in the outpatient setting. Therefore, application of most of the scores is limited in the preoperative setting. The ideal score to predict POPF would have to include variables that are obtainable pre- and intraoperatively, and also be reproducible in order to implement surgical or clinical strategies to avoid or reduce POPF-associated morbidity and mortality. It is therefore not surprising to find an association between a high concentration of amylase in drains and POPF. Risk stratification should begin preoperatively and can continue intraoperatively in order to allow for changes in surgical procedure such as drain placement, stenting of the pancreatic duct or proceeding to total pancreatectomy.

On univariate analysis, only four of the selected scores (Yamato et al. [21], Ansorge et al. [12], Mungroop et al. [18], Roberts et al. [19]) showed a significant association of all variables with POPF. Variables from these scores could be determined preoperatively by means of CT findings in addition to intraoperative data. However, the Callery et al. [14] fistula risk score is the most predominant. This score includes blood loss, which is difficult to adequately determine in time, when a change of surgical strategy might still be possible. The recently proposed alternative fistula risk score by Mungroop et al. [18] ultimately provides adequate stratification of our cohort using variables which could be determined preoperatively. Although it was not the aim of this study to create a new risk score for POPF, the ideal score to predict POPF in our study population would have to include histology type, PDD, postoperative CRP, and BMI.

Total pancreatectomy may reduce perioperative morbidity and mortality by eliminating the risk of POPF. It should therefore be considered a suitable treatment option in patients with high-risk pancreatic anastomosis, especially in the age of autologous islet cell transplantation [23].

In the management of POPF [24], it is important to take into consideration reconstruction techniques. While some institutions favor pancreaticogastrostomy (PG), at our institution, pancreaticojejunostomy (PJ) is routinely performed. However, there is still controversy about which technique is better for preventing POPF. A recent German multicenter randomized controlled trial demonstrated no significant difference in the rates of POPF in patients who underwent PG versus PJ [25]. In an Italian randomized study (The Verona Trial) [10] of patients at highest risk for developing POPF, patients who underwent either PG with externalized stent or PJ with externalized stent experienced similar rates of POPF of up to 50%. However, it was concluded that patients who underwent PG had a higher risk of morbidity. Some meta-analyses [26–28] have shown a reduced incidence of insufficiency of the PG compared to PJ. PJ has been shown to be more physiological, as reduced incidence of impaired glucose tolerance, steatorrhea, and atrophic changes of the remaining pancreas have been reported in patients with PJ compared to patients with PG [29–31]. Nevertheless, it is hypothesized to adhere to well established surgical techniques to achieve best performance.

Placement of drains appears to play an important role in the diagnosis and treatment of relevant POPF [9]. Drains are used therapeutically to evacuate pancreatic secretion and prevent autodigestion, which can lead to death in the case of vessel erosion. On the other hand, therapeutic lavage could be performed using selected drains. This helps to control bacterial flora by preventing abscess formation and diluting the pancreatic enzymes, thereby reducing the risk of autodigestion. There are many studies available on the application and management of drains [32, 33]. Using such measures in high-risk patients can reduce their risk of POPF complications.

There is some evidence that stent placement in the pancreatic duct for PJ drains trypsin distal to the anastomosis and supports healing of the latter, thereby reducing complications associated with POPF [34]. However, there are complications associated with both external and internal stent placement, including stent dislocation, excessive loss of digestive fluid, and ascending infection leading to cholangitis and subsequent liver abscess formation. Several RCTs have not shown a better performance in patients with stent placement for PJ compared to patients without stent with regard to incidence of POPF [34, 35]. However, it is still not clear whether stent implantation really helps to reduce morbidity associated with POPF.

Topical application of fibrin glue to the pancreatic anastomosis was thought to reduce POPF by sealing torn pancreatic tissue [36]. Most reports tend to suggest that this procedure has no effect whatsoever on POPF [37–39]. Similarly, omental wrapping techniques have also been evaluated and were thought to prevent POPF and associated bleeding. Although a reduction in intraabdominal complications was seen, there was no reduction in POPF [40, 41]. Octreotide and octreotide analogs inhibit pancreatic exocrine secretion [42] and are used by centers as prophylactic agents to prevent POPF after pancreatic resection. Although there is still some controversy regarding the efficacy of these agents [43], two RCTs showed a decrease in POPF in patients undergoing pancreatic head resection [44, 45]. A randomized trial from Finland showed that preoperative intravenous application of hydrocortisone was not inferior to pasireotide in patients undergoing partial pancreatectomy with regard to incidence of POPF [46].

In a recent publication synthesizing perioperative risk factors for POPF, special attention was given to factors usually overlooked in pancreatic surgery which might have an effect on healing of the pancreatic anastomosis such as duration of surgery, perfusion of the pancreas, hypotension episodes, and volume of fluid transfused. Furthermore, it was suggested that metabolism, inflammasome, and the microbiome may play a role in the complex mechanisms and interactions involved in the development of POPF [47].

On univariate analysis, a negative association was seen for history of weight loss, history of pancreatitis, low postoperative CRP, and increase in PDD. On multivariate analysis, only postoperative CRP was negatively associated with POPF. A number of studies have shown that an increase in postoperative CRP correlates with postoperative complications following pancreatic surgery and POPF [48, 49]. However, the diagnostic accuracy tends to be low to moderate [50]. This can be explained by an immune-mediated response to inflammation and subsequent infection.

Limitations of this study are its retrospective nature and missing data. However, we believe that missing data was not systematic but missing at random. Of note is the Braga et al. score, which primarily aimed to determine a score for postoperative complications. However, the major morbidity component was POPF, making the score adequate for predicting the risk of POPF. In addition, some of the older scores used the original definition of POPF, which included biochemical leak as a clinically relevant POPF, thus making the data difficult to interpolate.

We present data with a relatively high incidence of POPF, which might reduce the generalizability of the results. In order to reduce the incidence of POPF in our clinic, we created a standard operating procedure (SOP) for the management of patients planned for pancreatic head resection. This includes preoperative subcutaneous application of octreotide 100 µg in high-risk patients (patients with papillary tumors, tumors of the distal biliary tract, absence of pancreatic duct dilatation), preoperative biliary tract drainage in the absence of cholangitis and bilirubin level > 150 µg/l, and intravenous application of 100 mg hydrocortisone during intubation. During surgery, we recommend restrictive infusion therapy. The pancreaticojejunal anastomosis is performed with synthetic, resorbable, monofilament sutures made from polymer poly-p-dioxanone (PDS). The choledochojejunostomy is also performed using PDS single button sutures, 20 cm distal to the pancreaticojejunal anastomosis. The gastrojejunal anastomosis with preservation of the pyloric ring is sutured in two continuous rows using PDS 5–0 suture material, 50 cm distal to the choledochojejunostomy. With regard to drainage placement and the management of high-risk patients, we recommend using the fistula risk score proposed by Callery et al. High-risk patients also receive hydrocortisone and octreotide postoperatively. With this SOP, we hope to reduce the incidence of POPF in our clinic.

## Conclusion

We performed an analysis of the application of the selected scores using the number of citations for each of these scores in the literature to date in order to better establish our conclusion and found that the most cited score was the one by Callery et al. with 210 citations in PubMed. The scores of Braga et al. and Wellner et al. followed with approximately 60 citations each. The remaining scores were cited less than 50 times. The Mungroop et al. score is quite new and was only cited 21 times. However, this score is based on the Callery et al. score and excludes intraoperative blood loss, which was statistically non-significant in our analysis.

There are many pre-, intra-, and postoperative strategies available in the management of POPF. It is therefore of major importance to synthesize these strategies and apply them in selected high-risk patients undergoing pancreatic surgery. Correct stratification of patients is possible using most of the above risk scores. The authors of this publication believe that only a pre- and intraoperative risk stratification of patients is reasonable in order to apply additional salvage strategies during and after surgery to prevent POPF-associated morbidity and mortality. Furthermore, reducing the incidence of POPF through the selective implementation of mitigating strategies in high-risk patients could reduce POPF-related costs. The application of fistula risk scores is therefore reasonable. We suggest using the risk score proposed by Callery et al. and Mungroop et al. to predict POPF because they are easy to determine. In particular, the pre- and intraoperative availability of significant factors will help in making decisions relating to salvage strategies.

## Data Availability

The datasets generated and analyzed during the current study are available from the corresponding author on reasonable request.

## Code availability

The analysis code is available from the corresponding author on reasonable request.

## Abbreviations

ASA: American Society of Anesthesiologists
BMI: Body mass index
CRP: C-reactive protein
CT: Computed tomography
DPPHR: Duodenum-preserving pancreatic head resection
MDP: Main pancreatic duct
PC: Pancreatic consistency
PDD: Pancreatic duct diameter
PPPD: Pancreaticoduodenectomy
PG: Pancreaticogastrostomy
PJ: Pancreaticojejunostomy
PIS: Postoperative inflammatory score
POPF: Postoperative pancreatic fistula
PPH: Postpancreatectomy hemorrhage
RCT: Randomized controlled trial
ROC: Receiver operating characteristic
